# Insulin Resistance-Associated Interhemispheric Functional Connectivity Alterations in T2DM: A Resting-State fMRI Study

**DOI:** 10.1155/2015/719076

**Published:** 2015-04-30

**Authors:** Wenqing Xia, Shaohua Wang, Andrea M. Spaeth, Hengyi Rao, Pin Wang, Yue Yang, Rong Huang, Rongrong Cai, Haixia Sun

**Affiliations:** ^1^Medical School of Southeast University, No. 87 Dingjiaqiao Road, Nanjing 210009, China; ^2^Center for Functional Neuroimaging, University of Pennsylvania, 3710 Hamilton Walk, Philadelphia, PA 19104, USA; ^3^Department of Endocrinology, Affiliated Zhongda Hospital of Southeast University, No. 87 Dingjiaqiao Road, Nanjing 210009, China; ^4^Center for Sleep and Circadian Neurobiology, Perelman School of Medicine, University of Pennsylvania, 3710 Hamilton Walk, Philadelphia, PA 19104, USA

## Abstract

We aim to investigate whether decreased interhemispheric functional connectivity exists in patients with type 2 diabetes mellitus (T2DM) by using resting-state functional magnetic resonance imaging (rs-fMRI). In addition, we sought to determine whether interhemispheric functional connectivity deficits associated with cognition and insulin resistance (IR) among T2DM patients. We compared the interhemispheric resting state functional connectivity of 32 T2DM patients and 30 healthy controls using rs-fMRI. Partial correlation coefficients were used to detect the relationship between rs-fMRI information and cognitive or clinical data. Compared with healthy controls, T2DM patients showed bidirectional alteration of functional connectivity in several brain regions. Functional connectivity values in the middle temporal gyrus (MTG) and in the superior frontal gyrus were inversely correlated with Trail Making Test-B score of patients. Notably, insulin resistance (log homeostasis model assessment-IR) negatively correlated with functional connectivity in the MTG of patients. In conclusion, T2DM patients exhibit abnormal interhemispheric functional connectivity in several default mode network regions, particularly in the MTG, and such alteration is associated with IR. Alterations in interhemispheric functional connectivity might contribute to cognitive dysfunction in T2DM patients.

## 1. Introduction

Type 2 diabetes (T2DM) is associated with poor cognition, including memory and visuospatial ability [1, 2]. Brain function abnormalities may underlie the observed cognitive deficits in T2DM patients. Numerous studies have investigated the brain activity of T2DM patients using fMRI and observed a decrease in neuronal activity in bilateral middle temporal gyrus (MTG) [3], decreased functional connectivity between bilateral hippocampus and cerebral hemispheres [4], and bilateral atrophy in specific regions, such as the hippocampus and the prefrontal cortex [5] which likely contribute to the cognitive dysfunction of these patients. Recently, it was reported that T2DM patients exhibit reduced WM in CC [6] and widespread WM disruptions [7]. As the largest white-matter (WM) connection between the cerebral hemispheres, the CC is the morphological correlate of interhemispheric connectivity. Abnormality in the CC leads to cognitive decrements, such as attentional function and semantic activation [8, 9]. Interhemispheric functional connectivity deficits may therefore play an important role in the cognitive dysfunction [10–12]. Taken together, in the brain of T2DM patients, interhemispheric functional connectivity abnormalities may occur.

Interestingly, insulin resistance (IR), the hallmark symptom of T2DM, has been associated with brain functional connectivity changes in T2DM patients the brain; pathogenesis might be mediated by insulin resistance (IR) [13, 14]. A recent resting-state functional magnetic resonance imaging (rs-fMRI) study provided direct evidence that IR, which is a main T2DM characteristic, is associated with reduced functional connectivity in T2DM patients; IR might reflect the functional changes in the brain of these patients [15]; however, whether or not IR relates to altered interhemispheric connections is unknown.

Voxel-mirrored homotopic connectivity (VMHC) is a novel approach that directly measures the interhemispheric resting-state functional connectivity (RSFC). In other words, it is a voxel-wise measurement of functional homotopy and reveals the synchrony of RSFC between a voxel in one hemisphere and its mirrored counterpart in the other [16]. Compared with most analytical techniques that focus on functional connectivity, computing VMHC in the entire brain does not require a priori assumption, namely, choosing the regions of interest. Thus, VHMC results are comprehensive. In previous studies, patients with cocaine addiction, schizophrenia, and depression have been evaluated using the VMHC method [17–19]. To our knowledge, T2DM-related alterations in interhemispheric functional connectivity between the cerebral hemispheres have not been studied previously.

In the current study, we assessed homotopic RSFC using the VMHC approach in order to determine the existence of abnormal interhemispheric functional connectivity in T2DM patients. In addition, we examined whether interhemispheric functional connectivity related to cognitive performance. Finally, given that IR may participate in the cognitive decline related to diabetes, we examined whether IR related to interhemispheric functional connectivity in T2DM patients.

## 2. Materials and Methods

### 2.1. Participants

All individuals provided a written informed consent before their participation in the study, which was approved by the Research Ethics Committee of the Affiliated Zhongda Hospital of Southeast University. This research was conducted in accordance with the Declaration of Helsinki (2008) of the World Medical Association.

This study was conducted from June 2012 to December 2013. A total of 62 subjects, including 32 diabetic patients and 30 healthy subjects, participated. The diabetic and healthy subjects were matched based on their sex, age, body mass index (BMI), and educational level. All subjects were right handed and completed at least 6 years of education. Diabetic patients met the diagnosis of T2DM according to the World Health Organization 1999 criteria [20]. The patient age range was 45 years to 70 years, with an average age of 59.5 ± 8.2 years. Patients had been diagnosed with diabetes for 3 to 20 years (mean = 10 ± 5.8 years) and were on various oral hypoglycemic agents without any insulin-sensitizing meditations. No hypoglycemic events were recorded.

Patients with a history of known stroke, alcoholism, head injury, Parkinson's disease, epilepsy, major depression, other neurological or psychiatric illness, major medical illness (e.g., cancer, anemia, and thyroid dysfunction), and severe visual or hearing loss were excluded from the study. In addition, patients with macrovascular diseases (e.g., cerebrovascular and cardiovascular diseases) or clinically detectable microvascular complications (e.g., retinopathy, nephropathy, and neuropathy) were also excluded.

### 2.2. Clinical Data and Neuropsychological Test Information

The general information and sociodemographic characteristics of all subjects were collected. Blood samples were obtained at 8 A.M., after an overnight fast, via venipuncture to assess the levels of fasting blood glucose, fasting serum insulin, HbA1c, and blood lipid content. IR index [homeostatic model assessment-insulin resistance (HOMA-IR)] was calculated using the following formula:

(1) $$\begin{array}{c} \frac{\text{fasting glucose}\left(\text{mmol}/\text{L}\right)\times \text{fasting insulin}\left(\mu \text{U}/\text{mL}\right)}{22.5}. \end{array}$$

To acquire information on participants' general cognitive function, memory, attention, executive function, and visuospatial skills, patients and healthy controls underwent a series of neuropsychological tests, including Mini Mental State Exam (MMSE), Montreal Cognitive Assessment (MoCA), Auditory Verbal Learning Test (AVLT), Digit Span Test (DST), Trail Making Test-A and B (TMT-A and TMT-B), Clock Drawing Test (CDT), and Hamilton Depression Scale (HAMD). Generally, all the tests were performed in a fixed order and took 40 minutes to complete. An experienced neuropsychiatrist facilitated the testing process, and a single-blind method was used.

### 2.3. MRI Acquisition

All participants with relatively stable glucose levels underwent the MRI scan at 11 a.m. on the day clinical data and neuropsychological tests were conducted. Caffeine intake was prohibited. Imaging data were acquired using a 3.0T MRI scanner (Siemens MAGENETOM Trio) with a birdcage head coil. Earplugs were used to reduce the scanner noise. The subjects lay supine with their head held in place by foam pads and a belt to minimize motion. They were instructed to lie quietly with their eyes closed without falling asleep, not think of anything in particular, and avoid any head motion during fMRI. Fluid-attenuated inversion recovery scans were also acquired: TR = 8,500 ms, TE = 94 ms, slices = 20, thickness = 5 mm, and voxel size 1.3 × 0.9 × 5 mm^3^. Functional images were collected axially using an echo-planar imaging sequence as follows: repetition time (TR) = 2000 ms, echo time (TE) = 25 ms, slices = 36, thickness = 4 mm, gap = 0 mm, field of view (FOV) = 240 × 240 mm^2^, acquisition matrix = 64 × 64, and flip angle (FA) = 90°. High-resolution 3D T1-weighted axial images covering the entire brain were acquired using the following parameters: TR = 1900 ms, TE = 2.48 ms, slices = 176, thickness = 1 mm, gap = 0 mm, FA = 90°, acquisition matrix = 256 × 256, and FOV = 250 × 250 mm^2^. For each individual, the entire process lasted for 744 seconds.

### 2.4. Image Preprocessing

Analyses were conducted with Data Processing Assistant for rs-fMRI (DPARSF) programs [21], based on statistical parametric mapping (SPM8, http://www.fil.ion.ucl.ac.uk/spm) and rs-fMRI data analysis toolkits (REST, http://www.restfmri.net). The fMRI sequence was scanned interleaved. A total of 240 volumes were scanned, and the first 10 volumes were discarded to allow for the signal equilibrium of the initial magnetic resonance signals and the adaptation of the subjects to the circumstances. The remaining 230 consecutive volumes were used for data analysis. Subsequently, the following procedures were conducted in order: slice-timing adjustment, realignment for head-motion correction, spatial normalization to the Montreal Neurological Institute (MNI) template (resampling voxel size = 3 × 3 × 3 mm^3^), and smoothing with an isotropic Gaussian kernel (FWHM = 4 mm), detrend, and filtering (0.01–0.08 Hz).

We excluded the following subjects from the study: 2 healthy control and 2 T2DM patients with poor image qualities and head movement exceeding 2.0 mm of maximum translation in any of the *x*, *y*, and *z* directions or 2.0° of the maximum rotation around the three axes. Thus, further analysis was conducted on 30 patients and 28 healthy controls.

### 2.5. Interhemispheric Correlation

The VMHC computation was processed using REST software. As described in the previous study [16], the homotopic RSFC was computed as the Pearson's correlation coefficient between the residual time series of each voxel and that of its symmetrical interhemispheric counterpart. Afterwards, the correlation values were Fisher *z*-transformed to improve the normality of the values. The resultant values were referred to as the VMHC and were used for the group analyses.

### 2.6. Structural Image Analysis

To eliminate the effect of structural damage on VMHC measurements, we performed a voxel-based morphometry (VBM) approach to estimate gray matter (GM) brain volumes using the VBM8 toolbox (http://dbm.neuro.uni-jena.de/vbm) running in the Statistical Parametrical Mapping 8 (http://www.fil.ion.ucl.ac.uk/spm/) software on MATLAB 7.10.0. In the preprocessing step of the VBM, DARTEL was used to improve intersubject registration of the structural images. Briefly, cerebral tissues were segmented into GM, WM, and cerebrospinal fluid using a unified segmentation algorithm [22]. T1 magnetic resonance images were normalized to the MNI template using affine linear registration, followed by Gaussian smoothing (FWHM = 8 mm). Finally, the resulting voxel-wise GM volume maps were entered as covariates in the functional data analysis.

### 2.7. Statistical Analysis

*Demographic and Clinical Characteristics Analysis.* Differences in demographic, clinic, and neuropsychological test scores between the patients and the healthy controls were analyzed using between-group *t*-test for the means and *χ* ^2^-test for the proportions (*p* < 0.05 was considered statistically significant).

*VMHC Analysis*. Individual VMHC maps were entered into a voxel-wise two-tailed *t*-test to examine the differences in interhemispheric functional connectivity between groups. The modulated GM maps obtained from the VBM analysis were applied to exclude the possible effects of structural differences. Factors such as age, sex, BMI, level of education, cerebral signals (global mean signal, WM signal, and cerebrospinal fluid signal), and motion parameters were included as nuisance covariates to control the possible effects of these factors on the results. The result was also determined via multiple comparison correction using the AlphaSim program (http://afni.nimh.nih.gov/pub/dist/doc/manual/AlphaSim.pdf) determined by Monte Carlo simulation (parameters: single voxel *p* value = 0.05, a minimum cluster size of 85 mm^3^, FWHM = 4 mm, within a gray matter mask corresponding to the AAL atlas).

*Correlation Analysis*. HOMA-IR was transformed into natural logarithm to reduce the positive skew in the distribution. To investigate the relationship between the VMHC and neurocognitive performance, we extracted the mean VMHC values in each region which shows significant alterations between groups and evaluated the partial correlation coefficients between the interhemispheric functional connectivity and each neurocognitive test as well as with log HOMA-IR in a voxel-wise manner. The relationships between the log HOMA-IR and the neuropsychological tests were also calculated. The correlations were corrected for age, sex, BMI, and educational level. A *p* value of less than 0.05 was considered statistically significant; Bonferroni correction was used for multiple comparisons.

## 3. Results and Discussion

### 3.1. Clinical and Neuropsychological Data

Demographic and clinical characteristics of the T2DM patients and the healthy controls are summarized in Table 1. Cognitive results are presented in Table 2. The groups did not differ significantly in terms of age, sex, education level, smoking history, BMI, blood pressure, and blood lipid content. As expected, HbA1c, fasting glucose, fasting serum insulin, and HOMA-IR index of the patients were elevated (all *p* < 0.001). The two groups did not differ in terms of blood pressure- and cholesterol-lowering medication intake. In terms of cognitive performance, T2DM patients scored lower then healthy controls on AVLT-immediate recall, TMT-B, and DST (*p* < 0.05), whereas the other neuropsychological tests showed slight decreases in cognitive performance, but the differences were insignificant (*p* > 0.05).

### 3.2. Structural Results

The GM, WM, and brain parenchyma volumes of the two groups were not significantly different, but the indices of the T2DM patients were slightly lower than those of the healthy controls (Table 3).

### 3.3. Interhemispheric Connectivity Differences between Groups

**Figure 1:**
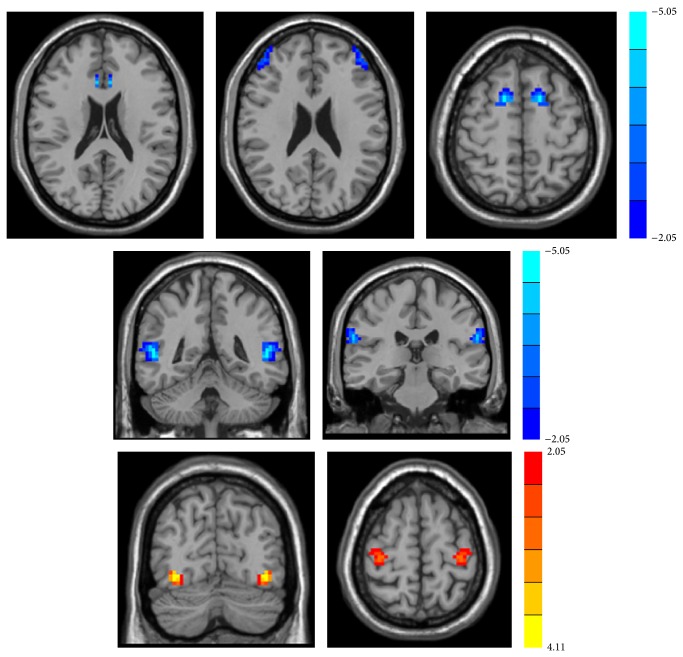
The regions which showed significantly decreased VHMC values between groups. Thresholds were set at a corrected *p* < 0.05, determined by Monte Carlo simulation.

The T2DM patients showed significantly lower interhemispheric connectivity in several brain regions, including MTG, middle frontal gyrus, superior frontal gyrus (SFG), inferior parietal lobule, and anterior cingulate gyrus than the healthy controls. Increased interhemispheric functional connectivity was observed in the inferior occipital gyrus and precentral gyrus (Figure 1 and Table 4).

### 3.4. Correlation Analysis Results

**Figure 2:**
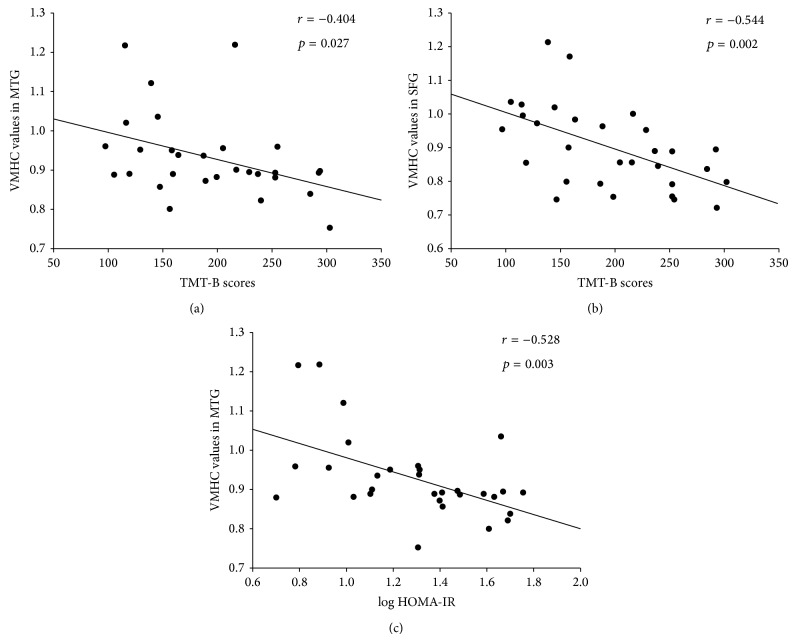
(a) Correlation between TMT-B score and mean VMHC values in middle temporal gyrus (*r* = −0.404, *p* = 0.027) in diabetic patients. TMT-B, Trail Making Test-B. (b) Correlation between TMT-B score and mean VMHC values in superior frontal gyrus (*r* = −0.544, *p* = 0.002) in diabetic patients. (c) Correlation between HOMA-IR and mean VMHC values in middle temporal gyrus (*r* = −0.528, *p* = 0.003, resp.) in diabetic patients. HOMA2-IR, homeostasis model assessment-insulin resistance.

Interhemispheric connectivity in the MTG and SFG were inversely correlated with performance on TMT-B (*r* = −0.404, *p* = 0.027; *r* = −0.544, *p* = 0.002, resp.) among T2DM patients (Figures 2(a) and 2(b)). However, the former significant correlation did not remain significant after Bonferroni correction. Log HOMA-IR was negatively correlated with the interhemispheric connectivity in the MTG among T2DM patients (*r* = −0.528, *p* = 0.003) (Figure 2(c)). Such correlation was maintained even after Bonferroni correction. Similarly, correlation analysis of the other regions which showed differences in interhemispheric connectivity between groups with log HOMA-IR and cognitive tests were also performed; however, no significant relationships were observed. No correlation was detected between log HOMA-IR and neuropsychological tests. We observed that HOMA-IR was positively associated with TMT-B scores, but this association was not statistically significant (*p* > 0.05).

### 3.5. Discussion

Interhemispheric connectivity was significantly reduced in several brain regions in T2DM patients compared to healthy control subjects, and decreased interhemispheric in the MTG was related to IR in T2DM patients. Although T2DM patients and healthy controls did not differ in terms of global cognitive function, evaluated by MMSE and MoCA, T2DM patients exhibited cognitive impairment on tasks of memory, attention, and executive function. TMT-B performance, an assessment involved in frontal functioning, negatively correlated with interhemispheric connectivity in the MTG and SFG of T2DM patients, a finding that is consistent with previous study results [3, 23, 24]. The significant correlation failed to persist after multiple comparisons correction, which may be partly due to the relatively strict method of testing. Contrary to previous studies, we did not observe structural volumes differences between healthy control subjects and T2DM patients [25, 26]. The differences in inclusion and exclusion criteria, disease duration, and age ranges may underlie this discrepancy. In addition, the patients in this study did not show any severe or chronic complications associated with T2DM.

The attenuated interhemispheric connectivity was shown in distributed regions, most of which were within the default mode network (DMN). These brain regions have also been partly observed in recent diffusion tensor imaging studies [27, 28]. Interestingly, aberrant interhemispheric connectivity in MTG was negatively correlated with HOMA-IR, which indicated the degree of IR. Musen et al. validated that T2DM patients showed reduced functional connectivity between the posterior cingulate cortex and multiple DMN regions, including the MTG [15]. The sample was enlarged in our previous work, and results implied that the altered spontaneous neuronal activity in the brain of T2DM patients was mainly observed in the bilateral MTG region, which was correlated with cognitive dysfunction and ß-cell function [3].

Numerous studies have confirmed the importance of insulin and IR in cognition [29–31]. Rotte et al. demonstrated that insulin enhances neuronal activity within the medial temporal lobe [32]. Similarly, the negative association between IR and DMN-hippocampal functional connectivity was examined previously [33]. IR leads to altered glucose transport into the brain and/or into hippocampal cells and then results in hippocampus damage and reduced memory performance [34]. In the current study, however, IR was not significantly correlated with cognitive performance. Especially, we observed that HOMA-IR was positively associated with TMT-B scores, but this association was not statistically significant. This may result from the limitations of this study, such as small sample size and some other potential confounding factors. Given the significant indirect effect of HOMA-IR→interhemispheric connectivity→TMT-B scores, we suggested that instead of directly affecting cognition, IR might be relevant to the cognitive function of the T2DM patients by disturbing the interhemispheric connectivity in MTG, and such activity could be detected via fMRI. The associations require further verification, and the results in the current provide enlightenment for future investigations.

Patients with T2DM exhibited increased interhemisphere connectivity in two brain areas, including inferior occipital gyrus and precentral gyrus. Increased interhemispheric functional connectivity in such regions may represent a compensatory mechanism to recruit additional neural resources to attenuate cognitive decline. The inferior occipital gyrus participates in the rearrangement and deployment of attentional resources [35]. The precentral gyrus is typically spared in the advanced stages of AD [36]. In general, the hyperactivity located in these regions may participate in alleviating cognitive disability in T2DM patients. However, we did not detect a significant correlation between interhemispheric connectivity in these regions with cognitive performance. Thus, the details of this kind of mechanism remain unclear. The mechanism may be related to the loss of inhibiting neurons [37] or the loss of connectivity in other parts of the brain [38]. Further studies are needed to assess this compensatory hypothesis.

This study has several limitations. First, we provided a relatively small sample size in this cross-sectional study. Thus, cause-and-effect relationships cannot be ascertained. The limited population size also hindered stratification based on disease duration. Second, several potential confounding factors, such as undiagnosed microangiopathy and ApoE*ε*4 genotype, could not be accounted for and may affect various cognition and clinical variables. Third, the MoCA scores of the healthy controls in this study were abnormal, which is an inherent flaw of the study. Finally, therapeutic regimens of the individuals were variable and therefore we cannot control what effects this medication had on the outcome variables in the study. For instance, Metformin and Rosiglitazone may affect the cognitive function of diabetic patients [39–41].

## 4. Conclusions

Patients with T2DM experience altered interhemispheric connectivity in several brain regions, particularly in the MTG. In addition, altered interhemispheric connectivity was negatively correlated with IR in patients with T2DM. Altered interhemispheric connectivity may contribute to the cognitive dysfunction exhibited by T2DM patients. Future studies are needed to determine the role of IR in such changes. The current study implies the importance of interhemispheric connectivity as a target for interventions and/or therapeutic targets related to the neuropathology and cognitive decrement associated with T2DM.

## Figures and Tables

**Table 1:** Demographic and clinical characteristics.

Items	T2DM patients (*n* = 30)		Healthy controls (*n* = 28)		*p* value
	Mean	SD	Mean	SD	
Age, years	59.5	8.2	56.2	7.1	0.106
Gender, male : female	16 : 14		13 : 15		0.599
Education levels, years	9.8	3.8	10.6	3.2	0.299
Diabetes duration, years	10.0	5.8	—	—	—
History of smoking, yes : no	4 : 26		6 : 22		0.415
BMI, kg/m^2^	25.3	2.7	24.7	2.4	0.329
Systolic BP, mmHg	129.0	19.7	129.5	15.0	0.895
Diastolic BP, mmHg	78.4	9.4	80.5	7.4	0.342
Hb_A1c_, % (mmol/mol)	8.0 (64)	1.6 (17.5)	5.8 (40)	0.6 (6.6)	<0.001^∗^
Fasting glucose, mmol/L	8.3	2.4	6.0	0.9	<0.001^∗^
Fasting serum insulin, *μ*U/L	11.0	4.0	5.6	2.1	<0.001^∗^
HOMA-IR	3.8	1.1	1.5	0.5	<0.001^∗^
Triglyceride, mmol/L	1.5	0.7	1.4	0.7	0.814
Total cholesterol, mmol/L	5.5	1.2	5.7	0.8	0.576
LDL-C, mmol/L	3.4	0.8	3.4	0.5	0.996
HDL-C, mmol/L	1.4	0.3	1.4	0.3	0.987
Intima-media thickness, mm	1.0	0.2	0.9	0.2	0.059
Blood pressure lowering medications, yes : no	10 : 20		8 : 20		0.695
Cholesterol lowering medications, yes : no	9 : 21		3 : 25		0.07

^*^ *p* < 0.05.

HOMA2-IR, homeostasis model assessment-insulin resistance; LDL-C, low-density lipoprotein cholesterol; HDL-C, high-density lipoprotein cholesterol.

**Table 2:** Cognitive scores and depressive symptoms.

Items	T2DM patients (*n* = 30)		Healthy controls (*n* = 28)		*p* value
	Mean	SD	Mean	SD	
MMSE	28.7	1.1	29.1	1.3	0.196
MoCA	23.0	3.0	24.4	2.1	0.053
AVLT-immediate recall	18.6	4.5	20.6	2.9	0.046^∗^
AVLT-delayed recall	6.5	2.6	6.7	1.7	0.718
TMT-A	73.0	22.1	67.6	17.0	0.306
TMT-B	195.2	62.3	159.1	43.4	0.014^∗^
CDT	3.2	0.8	3.5	0.5	0.111
DST	10.9	1.9	12.2	2.4	0.028^∗^
HAMD	1.3	1.1	1.2	1.1	0.773

^*^ *p* < 0.05.

MMSE, Mini Mental State Exam; MoCA, Montreal Cognitive Assessment; AVLT, Auditory Verbal Learning test; TMT, Trail making test; CDT, Clock drawing test; DST, Digit span test; HAMD, Hamilton Depression Scale.

**Table 3:** Comparisons of the brain volumes between groups.

Brain volume	T2DM patients (*n* = 30)		Healthy controls (*n* = 28)		*p* value
	Mean	SD	Mean	SD	
Gray matter	578.6	21.6	585.3	29.1	0.323
White matter	529.4	23.1	531.3	25.1	0.770
Brain parenchyma	1108.0	36.2	1116.6	34.8	0.363

**Table 4:** Regions showing significant differences in VMHC between patients and healthy controls.

Brain regions	MNI coordinates *x*, *y*, *z* (mm)	Peak *t* score	Voxels
Decreased in T2DM patients			
Middle temporal gyrus	±54, −45, 6	−4.8383	289
Middle frontal gyrus	±45, 45, 24	−2.0195	100
Superior frontal gyrus	±15, 9, 57	−5.1613	267
Inferior parietal lobule	±63, −27, 21	−4.3842	97
Anterior cingulate gyrus	±6, 24, 21	−3.8309	91

Increased in T2DM patients			
Inferior osccipital gyrus	±36, −72, −9	4.7328	199
Precentral gyrus	±36, −27, 63	3.2897	234

A corrected threshold of *p* < 0.05 determined by Monte Carlo simulation was taken as meaning that there was a significant difference between groups. MNI: Montreal Neurological Institute; cluster size is in mm^3^.
